# Exposure to *Toxoplasma gondii* in the Roma and Non-Roma Inhabitants of Slovakia: A Cross-Sectional Seroprevalence Study

**DOI:** 10.3390/ijerph15030408

**Published:** 2018-02-27

**Authors:** Daniela Antolová, Martin Janičko, Monika Halánová, Peter Jarčuška, Andrea Madarasová Gecková, Ingrid Babinská, Zuzana Kalinová, Daniel Pella, Mária Mareková, Eduard Veseliny, HepaMeta Team

**Affiliations:** 1 Department of Parasitic Diseases, Institute of Parasitology SAS, Hlinkova 3, 040 01 Košice, Slovakia; antolova@saske.sk; 2 Faculty of Medicine, P. J. Šafárik University in Košice, Trieda SNP 1, 040 11 Košice, Slovakia; martin.janicko@gmail.com (M.J.); monika.halanova@upjs.sk (M.H.); peter.jarcuska@upjs.sk (P.J.); andrea.geckova@upjs.sk (A.M.G.); ingrid.babinska@upjs.sk (I.B.); zuzana.kalinova@upjs.sk (Z.K.); daniel.pella@upjs.sk (D.P.); maria.marekova@upjs.sk (M.M.)

**Keywords:** *Toxoplasma gondii*, Toxoplasmosis, seroprevalence, Roma people, Slovakia

## Abstract

The lifestyle, health and social status of the Roma are generally below the standards characteristic for the non-Roma population. This study aimed to find out the seropositivity to *Toxoplasma gondii* (*T. gondii*) in the population of Roma living in segregated settlements and to compare it with the prevalence of antibodies in the non-Roma population from the catchment area of eastern Slovakia. The seroprevalence of antibodies to *T. gondii* was significantly higher in the Roma group (45.0%) than in non-Roma inhabitants (24.1%). A statistically significant difference was also recorded between the two non-Roma groups in the study, 30.4% of those from the catchment area and 19.7% from the non-catchment area were seropositive. Univariate logistic regression confirmed poverty and higher age to be significant risk factors influencing the seropositivity to *T. gondii*. Of the clinical symptoms analyzed in the study, only muscle and back pain were associated with seropositivity to *T. gondii*. The close contact of Roma with an environment contaminated by different infectious agents and the insufficient hygiene, lower level of education, poverty, lack of water and household equipment and high number of domestic animals increase the risk of infectious diseases in the Roma settlements and subsequently the spread of communicable diseases at the national or even international level.

## 1. Introduction

*Toxoplasma gondii* (*T. gondii*) is an obligate intracellular protozoan parasite that infects all warm-blooded animals and has worldwide occurrence. In definitive hosts, members of the Felidae family, it has a sexual cycle, while in intermediate hosts it proliferates in a two-stage asexual cycle. The parasite is of medical and veterinary importance, as it can cause abortions and congenital diseases in infected intermediate hosts. *T. gondii* infection is common in humans, but the majority of cases in immunocompetent persons are asymptomatic, or various mild symptoms can be observed. If first contracted during pregnancy, *T. gondii* can be transmitted to the fetus and may cause abortion, neonatal death or fetal abnormalities [1]. Infection can be acquired by ingestion of infectious oocysts from the environment and water or by consumption of tissue cysts contained in raw or undercooked meat of infected intermediate hosts. It is suggested that the prevalence of toxoplasmosis in different countries depends mainly on the eating habits of the population. The handling and consumption of raw or undercooked meat are considered a health risk especially for pregnant women and immunosuppressed patients [2,3].

The Roma population is considered one of the largest minorities in Central and Eastern Europe [4,5]. In Slovakia, 1.97% of inhabitants declare themselves as being of Roma nationality, but the estimated real number exceeds 7.3%, with approximately one-sixth of them living in segregated settlements [6]. The lifestyle, health and social status of the Roma differ from the majority population. Their socioeconomic and health status as well as their education level are below the standards characteristic for the non-Roma population, implying also poverty and a lower quality of life [7,8]. Moreover, increased mortality and worse health status have been reported [5,9,10].

The mentioned features are much more obvious in segregated settlements, often characterized by illegal huts (often built from waste materials) and limited access to electricity, tap water and a sewerage system [11]. For settlements, a large concentration of humans and domestic animals is characteristic, and very often only a single well or stream serve as a water source for all residents.

Although the Roma ethnic minority is the most numerous in Europe, there are not many studies dealing with their health conditions and the occurrence of diseases [12,13], and data on the occurrence of parasitozoonoses in Roma are very scarce. Since significant differences between the lifestyle of the Roma and non-Roma populations have been described, a higher incidence of parasitic diseases in the Roma is assumed. The aim of this cross-sectional study was to assess the seropositivity to *T. gondii* in the Roma inhabitants of segregated settlements and to compare it with the seropositivity of the non-Roma population of eastern Slovakia. The way of life in the close vicinity of segregated settlements can influence the risk of infection. Therefore, the occurrence of antibodies in the non-Roma inhabitants living near Roma settlements (catchment area) and outside of these areas (non-catchment area) was compared. The occurrence of clinical symptoms and risk factors for infection were also analyzed.

## 2. Material and Methods

### 2.1. Data and Samples

In this study, data from the HepaMeta project performed in Slovakia in 2011 were used. The methodology for this cross-sectional population-based study using a community-based approach was previously published by Madarasová Gecková et al. [14]. The studied population involved Roma inhabitants of 10 different segregated communities in eastern Slovakia, and the non-Roma majority population from the same region was used as a control group. Only adult respondents (18–55 years old) without clinical signs of any acute disease were included in the study.

Altogether, 420 Roma respondents from settlements and 386 respondents from the majority population were sampled. The way of life in the close vicinity of segregated settlements can influence the risk of infection of inhabitants through the contamination of the environment by propagational stages of different parasitic agents. Therefore, the non-Roma respondents were divided into two groups. The first one comprised 158 persons living in rural areas in the vicinity of segregated settlements (catchment area), and the second group was composed of 228 inhabitants living in the areas outside of segregated Roma communities (most often in cities), without a Roma population (non-catchment area).

In addition to blood samples and selected medical information, questionnaires were obtained from all respondents included in the study. The questionnaires comprised data about the socio-demographic status, education, economic situation, living conditions and health of respondents. The questionnaires also included data about the presence of clinical symptoms (headache, abdominal pain, back pain, muscle pain, influenza-like symptoms, cough, diarrhoea, allergy, fatigue, insomnia, anxiety or stress); moreover, some clinical data were obtained during the collection of blood samples (height, weight, blood pressure) and further analyses of blood (biochemical markers etc.). For consecutive analyses, the level of education was assessed as a categorical variable containing three categories: elementary, vocational school and higher education. The economic situation was evaluated by the presence of problems with covering common living costs (rent, energy, health care, shopping costs and loans), and living conditions by the availability of sanitary equipment in households, the type of house and the form of in-house heating.

Approval for the study was obtained from the Ethics Committee of the Faculty of Medicine at Šafárik University in Košice (code 104/2011; approved 12 January 2011). Participation in the study was voluntary and anonymous and informed consent was obtained prior to the medical examination.

### 2.2. Determination of Anti-Toxoplasma gondii Antibodies

Sera were obtained from venous blood and stored at −20 °C until tested. Sandwich ELISA test kits EIA *Toxoplasm*a IgG (Test-Line Clinical Diagnostics Ltd., Brno, Czech Republic) were used to evaluate serum samples for the presence of specific IgG antibodies to *T. gondii*. The index of positivity (IP) was calculated for each sample according to the formula: “IP = sample OD (optical density)/average cut-off OD”. Samples with IP >1.1 were considered positive.

### 2.3. Statistical Analyses

Categorical variables are presented as relative frequency with 95% CI (confidence interval). Differences between categorical variables were analyzed using the Chi-square test, in 2 × 2 contingency tables; odds ratios (OR) and relative risk (RR) with 95% CI are also presented. Differences between the continuous variables were analyzed using the Mann-Whitney test. The risk profile was evaluated with a logistic regression model that also included adjustment for confounders (age and sex were unadjusted, other risk factors were adjusted for age and sex). A two-sided *p* value < 0.05 was considered statistically significant.

## 3. Results

Analysis of baseline parameters of the involved populations revealed significant differences between the Roma and non-Roma population of Slovakia (Table 1). We observed a significantly lower number of male participants in the analyzed Roma minority. Unemployment was significantly more common in Roma participants, and they also had achieved a lower education level than participants from the majority population. The Roma people lived significantly less frequently in brick houses, and the lack of basic household equipment and inability to cover common living expenses (poverty) were also more frequent in this minority. The average age of the analyzed groups was 33.2 ± 17.0 years among Roma, 27.9 ± 30.6 in non-Roma inhabitants from the catchment area and 32.3 ± 10.8 years in the majority population from the non-catchment area of eastern Slovakia.

Altogether, 806 persons were tested for the presence of anti-*Toxoplasma gondii* IgG antibodies. The analyzed groups comprised 420 Roma and 386 non-Roma participants. The seropositivity recorded in the Roma group (45.0%; 95% CI 40.3–49.8) was significantly higher (*p* < 0.0001; X = 37.7) than in non-Roma inhabitants (24.1%; 95% CI 20.9–28.6). A statistically significant difference (*p* < 0.05; X = 5.2) was also recorded between both non-Roma groups: 30.4% (95% CI 23.7–37.9) persons from the catchment area and 19.7% (95% CI 15.1–25.4) from the non-catchment area were seropositive. The relative risk of seropositivity to *T. gondii* in Roma was higher than in non-Roma inhabitants (2.28, 95% CI 1.72–3.02), and the OR value reached 3.33 (95% CI 2.28–4.86). Higher RR and OR were also confirmed in the group of majority inhabitants from the catchment area in comparison with those from the non-catchment area (OR = 1.77, 95% CI 1.11–2.84; RR = 1.54, 95% CI 1.08–2.19). All comparisons are referenced to the non-Roma participants living in the non-catchment area.

### 3.1. Factors Associated with Seropositivity to Toxoplasma gondii

Seroprevalence of toxoplasmosis varied with gender, age, education level, employment rate and payment problems of persons involved in the study. The prevalence of antibodies according to the analyzed groups of participants is presented in Table 2.

Risk factors for seropositivity to *T. gondii* were analyzed separately for Roma and both non-Roma groups. Poverty and higher age were found to be significant risk factors influencing the seropositivity to *T. gondii* in Roma, while gender, level of education, unemployment, living in non-brick houses and the number of people in a house were not significant risks for the prevalence of antibodies (Table 3).

### 3.2. Clinical Symptoms

In seropositive persons (all Roma and non-Roma respondents grouped together) the presence of clinical symptoms that could be related to *T. gondii* infection was studied. Of the symptoms included in the questionnaires, only muscle and back pain were associated with seropositivity. The presence of some psychological or neurological signs that could indicate some behavioral changes did not differ between the study groups (Table 4).

## 4. Discussion

*Toxoplasma gondii* is a zoonotic parasite that occurs in animals and humans throughout the world. It is assumed that approximately 25–30% of the human population is infected, but the prevalence varies widely between countries and also between communities within the same country or region. In general, lower prevalence rates are observed in North America, Northern Europe and South-east Asia; higher positivity is found in the countries of Central and Southern Europe, and high prevalence rates are typical for Latin America and tropical African countries [15,16]. In the United States, the examination of 15,960 sera revealed 10.8% seropositivity to *T. gondii* [17]. In eastern and north-eastern China 13.79% of analyzed human samples were positive [18], while in France, the seroprevalence of toxoplasmosis among 15,108 pregnant women reached 43.8% [19]. In the presented study, altogether 35.19% of the examined participants were positive, but significant differences were observed between the Roma (45.0%) and non-Roma (24.1%) communities. A significant difference was also observed between the seropositivity of the non-Roma population living in the vicinity of segregated settlements (catchment area) and inhabitants from the non-catchment area outside the segregated Roma communities (30.4% and 19.7%, respectively). The observed differences correspond with the lifestyle of both the Roma and non-Roma population. The Roma minority is considered to be socio-economically disadvantaged as it has been described in previous studies [20,21]. Segregated settlements are often built on loose soils, and the lack of drinking water and sanitary facilities, with numerous waste pits, sewerage and landfills in an adjacent area are characteristic [22]. Less preventive care and a less healthy lifestyle were also recorded in Roma people [13,23].

The mentioned features were also observed in the Roma population analyzed in this study. Roma participants were significantly more often unemployed, had a lower educational level and more often lived in poverty. They also lived significantly less frequently in brick houses; the number of people in one house was significantly higher, and the lack of household equipment (sewerage system, water supply, flush toilet, bathroom or shower and/or electricity supply) was significantly more frequent than in non-Roma participants. By contrast, a better lifestyle and living conditions were documented in the analysis of the baseline study parameters in the non-Roma majority.

The majority of non-Roma respondents from the “catchment area” came from villages situated near Roma settlements, which could contribute to the more frequent occurrence of seropositivity to *T. gondii* in comparison with persons from the non-catchment area. On the other hand, higher seroprevalence of antibodies can also be connected with life in a rural environment and the closer contact of participants with animals and soil. In an older study performed in Slovakia, Studeničová et al. [24] recorded 22.1% seroprevalence of toxoplasmosis in pregnant women, with significantly higher positivity detected in women from rural areas in comparison with those living in cities.

Analysis of seroprevalence rates showed that positivity to *T. gondii* significantly increased with age. In Roma participants, the highest occurrence of antibodies was recorded in those older than 50 years, while in non-Roma groups, persons between 30 and 39 years old were most often positive. Studeničová et al. [24], who detected the highest seropositivity in women age 35–44 years, also observed similar results. Higher seropositivity recorded in older respondents can be connected with the continual growth in the number of positive people in the area. IgG antibodies to *Toxoplasma gondii* appear in the blood 1–2 weeks post-infection, and the highest levels are detected 6 months after the infection and persist at low levels for a long time, sometimes for life [25]. Therefore, the total number of seropositive people can grow with age. No statistical significance was confirmed between seropositivity and the level of completed education or employment. However, in Roma as well as in non-Roma groups, higher seroprevalence of antibodies was recorded in people who completed only elementary school, followed by those with vocational school and participants with high school or university; and unemployed people were positive more often than participants who were employed. Jones et al. [26] recorded a similar tendency, finding a significant correlation between seropositivity and education level and employment of analyzed populations. The level of completed education also influences the possibility of getting a job. A lower education level is often related to manual labor occupations, e.g., in agriculture or the building industry, which are connected with more frequent exposure to soil and thus involve higher risk of infection than people who do white-collar work.

In this study, the seroprevalence of toxoplasmosis was significantly related only to the poverty of the Roma. A correlation between poverty and the occurrence of antibodies to *T. gondii* was also recorded in the study of Jones et al. [26], with 27.8% seropositivity in the population below the poverty line and 21.5% seroprevalence in people at or above the poverty line. Poor socio-economic status is often associated with unemployment or badly paid jobs. Unemployed people often try to improve their living standard, and those who live in villages often grow vegetables or fruits and are thus in repeated contact with the soil, a known source of infection in many cases. Another possibility of acquiring infection is drinking water contaminated with *T. gondii* oocysts. Sporulated oocysts have been detected in ground water supplies [27], and several waterborne outbreaks of toxoplasmosis have been recorded throughout the world [28]. Recently, oocysts were detected in air samples, thus revealing air as a new transmission route of the infection [29]. As the majority (62.7%) of Roma participants from segregated settlement lived without basic household equipment (sewerage system, water supply, flush toilet, bathroom or shower) we can assume that they were in close contact with natural sources of water, which could be contaminated by *T. gondii* oocysts. *T. gondii* infection in immunocompetent humans is asymptomatic in more than 80% of cases [30]. Mild symptoms are occasionally observed, such as fever, malaise, lymphadenopathy, myalgia, asthenia or other nonspecific clinical signs. Severe manifestations, such pulmonary and multivisceral involvement, encephalitis, myocarditis, hepatitis or sepsis syndrome, can also occur but are very rare [1,16,31]. In this study, only muscle and back pain appeared significantly more often in seropositive persons than in negative ones, which corresponds with the occurrence of myalgia reported in the literature [30]. The presence of some neurological or psychological disturbances did not differ significantly between seropositive and seronegative persons.

People can become infected with *T. gondii* after the ingestion of oocysts from the environment or tissue cysts from raw or undercooked meat of infected intermediate hosts. The risk of soil contamination by oocysts depends on the number of infected cats in the adjacent area. Oocysts shed by infected cats can remain infectious in moist soil or sand for up to 18 months [30]. For segregated Roma settlements, high numbers of domestic carnivores, dogs and cats, are typical. These animals are nearly always unregistered; they live almost without any veterinary care or deworming and are fed with leftovers and household garbage [11,22]. It can be said that their life is almost the same as the life of stray dogs and cats. Therefore, the health status of such animals is often poor. Poor hygienic conditions and high numbers of wild rodents in segregated settlements support the possibility of *T. gondii* circulating between animals and subsequently increase the infection risk for humans. Improperly cooked food and insufficient hygiene during food preparation present other risk factors of infection. Although Roma usually cook their food and meat appropriately, the lack of tap water or even water sources in the settlements increases the infection risk through the contamination of hands and kitchen equipment by *T. gondii* tissue cysts. Contact with meat and consumption of raw/undercooked meat, raw vegetables and fruits, and the usage of uncontrolled water sources were associated with increased infection risk in the studies of Retmanasari et al. [32] and Zhang et al. [18]. Relating to meat, pigs are considered the most probable source of human infection [1], but other species are also often infected. In Brazil, 37.9% of 224 pigs from legal slaughterhouses were tested seropositive to *T. gondii,* and 14.1% of tissue samples were positive by PCR *(*Polymerase Chain Reaction) [33]. In Slovakia, 2.16% of 970 examined slaughtered pigs were found to be seropositive to *T. gondii*. Moreover, further molecular analyses confirmed the dominance of the virulent strain of genotype I (85.7%) in the country, while only 14.3% of animals were infected by the avirulent strain (genotype II) [34]. Studeničová et al. [24] revealed a higher seropositivity rate in meat industry workers (50.0%) in comparison with healthy blood donors (27.7%). The mentioned facts could contribute to higher seroprevalence of *T. gondii* in inhabitants of Roma segregated settlements, but also in non-Roma participants coming from rural areas close to Roma communities.

## 5. Conclusions

Roma live in close contact with an environment contaminated by different infectious agents, not only of parasitic, but also of bacterial or viral origin. Insufficient hygiene, a lower level of education, poverty, a lack of water and household equipment and a high number of domestic animals increase not only the risk of circulation of infectious diseases in Roma settlements, but also enhances the spread of communicable diseases at the national or even international level.

## Figures and Tables

**Table 1:** Baseline parameters of the study.

Parameter	N	Roma Population N (%)	Non-Roma Population N (%)			
			Together	*p*	Catchment Area	Non-Catchment Area
Male gender	328	146 (34.5%)	182 (47.1%)	0.002	70 (44.3%)	112 (49.1%)
Age	823	33.2 (±17.0)	32.2 (±15.3)	₋	27.9 (±30.6)	32.3 (±10.8)
Unemployed	798	374 (89.3%)	100 (25.9%)	<0.0001	55 (37.7%)	45 (19.7%)
Education level	798					
Elementary	₋	333 (79.3%)	9 (2.3%)	<0.0001	8 (5.3%)	1 (0.4%)
Vocational school	₋	70 (16.7%)	83 (21.5%)	0.08	49 (32.7%)	34 (14.4%)
Higher	₋	9 (2.1%)	294 (76.2%)	<0.0001	93 (62.0%)	201 (85.2%)
Lack of basic household equipment *	823	269 (62.7%)	76 (19.6%)	<0.0001	51 (32.3%)	25 (10.6%)
Payment problems **	782	205 (48.7%)	45 (11.6%)	<0.0001	31 (24.1%)	14 (6.4%)
Life in bricked house	790	399 (94.5%)	358 (92.7%)	<0.0001	140 (98.6%)	218 (100.0%)
Number of people in house	726	7.5 (±5.3)	2.3 (±2.4)	<0.0001	2.2 (±1.6)	2.3 (±3.8)

N—number of participants, %—percentage of the population; P—P for the difference between Roma and non-Roma population; * Aggregate of lacking at least one item of the following: sewerage system, water supply, flush toilet, bathroom or shower, electricity supply; ** Aggregate of issue to pay at least one item of the following: rent, loan payment, healthcare, energies, other expenses.

**Table 2:** Seropositivity to *Toxoplasma gondii* in relation to gender, age, education and employment.

Parameter	Roma Population			Non-Roma Population								
				Together			Catchment Area			Non-Catchment Area		
	N	%	95% CI	N	%	95% CI	N	%	95% CI	N	%	95% CI
Total seropositivity	420	45.0	40.3–49.8	386	24.1	20.1–28.6	158	30.4	23.7–37.9	228	19.7	15.1–25.4
Gender												
Men	146	43.8	36.1–51.9	182	26.1	20.2–33.0	70	31.4	21.7–43.1	112	22.7	15.8–31.5
Women	247	45.6	44.4–57.0	206	22.3	17.2–28.5	88	29.5	20.3–40.2	118	16.9	11.1–24.8
Age												
18–29	136	33.8	26.4–42.1	131	15.3	10.0–22.5	49	20.4	11.3–33.8	82	12.2	6.6–21.2
30–39	143	46.9	38.9–55.0	176	28.4	22.3–35.5	59	32.2	21.6–44.9	117	26.5	19.3–35.2
40–49	123	50.4	41.7–59.1	61	21.3	12.8–33.3	33	30.3	17.3–47.5	28	10.7	2.9–28.1
50 and more	14	78.8	51.7–93.2	9	66.7	35.1–88.3	9 *	66.7 *	35.1–88.3	0	0	₋
Education level												
Elementary School	333	47.1	41.9–52.5	9	77.8	44.3–94.7	8 *	87.5 *	50.8–99.9	1 *	0.0 *	0.0–77.7
Vocational School	70	35.7	25.5–47.4	83	27.7	19.2–38.2	49	34.7	22.9–48.7	34	17.6	8.0–33.9
Higher	9 *	22.2 *	5.3–25.2	294	20.4	16.2–25.4	93	22.6	3.7–33.0	201	19.4	14.5–25.5
Employment												
Employed	44	36.4	23.7–51.2	278	23.0	18.5–28.3	91	27.5	19.3–37.5	187	20.9	15.6–27.3
Unemployed	366	46.4	41.4–51.6	99	24.2	16.8–33.6	55	32.7	21.8–46.0	44	13.6	6.0–27.1
Payment problems												
Yes	209	51.7	44.9–58.4	45	11.7	-	31	19.6	-	14	6.1	-
No	211	38.4	32.1–45.1	306	22.9	18.5–27.9	107	28.0	20.4–37.2	199	20.1	15.1–26.4
Houses												
Non-Bricked	23	47.8	29.2–67.0	2	50.0	9.5–90.6	2 *	50.0 *	9.5–90.6	0	0	₋
Bricked	390	44.6	39.8–49.6	358	23.7	19.6–28.4	140	31.4	24.3–39.5	218	18.8	14.2–24.6

N—Number of examined; %—seropositivity; CI—confidence interval; * too low number of examined for accurate analyses.

**Table 3:** Risk factors of seropositivity to *Toxoplasma gondii* for Roma a non-Roma population.

Parameter	Roma Population			Non-Roma Population					
				Catchment Area			Non-Catchment Area		
	OR	95% CI	P	OR	95% CI	P	OR	95% CI	P
Male sex	0.93	0.62–1.39	0.726	1.09	0.55–2.16	0.798	1.44	0.75–2.78	0.275
Age (years)	1.01	1.00–1.02	0.004	1.00	0.99–1.01	0.041	0.99	0.96–1.01	0.037
Unemployed	1.52	0.79–2.90	0.207	1.28	0.62–2.66	0.5	0.56	0.23–1.46	0.244
Education level *									
Elementary	3.12	0.64–15.25	0.159	24.00	2.79–206.2	0.004	₋	₋	₋
Vocational school	1.94	0.38–10.08	0.428	1.82	0.85–3.91	0.124	0.97	0.37–2.52	0.944
Lack of household equipment *	1.07	0.72–1.59	0.738	1.23	0.60–2.51	0.578	1.50	0.56–4.06	0.422
Payment problems **	1.69	1.14–2.50	0.009	1.59	0.71–3.57	0.263	0.33	0.04–2.62	0.295
Life in bricked house	0.88	0.38–2.04	0.764	0.46	0.03–7.50	0.584	₋	₋	₋
Number of people in house ***	0.97	0.93–1.02	0.235	1.09	0.82–1.44	0.548	0.97	0.88–1.08	0.6

N/A not analyzed; CI—confidence interval; * Education—odds compared to “higher” education; ** Household equipment—aggregate of lacking at least one item of the following: sewerage system, water supply, flush toilet, bathroom or shower, electricity supply; ** Payment problems—aggregate of inability to pay at least one item of the following: rent, loan payment, healthcare, energies, other expenses; *** OR for 1 person increase.

**Table 4:** Occurrence of clinical symptoms in persons seropositive and seronegative to *T. gondii.*

Clinical Sign	Unadjusted Difference		
	Positive (%) (*n* = 282)	Negative (%) (*n* = 524)	*p*
Headache	67.9	62.8	0.12
Muscle and back pain	57.0	49.2	0.027
Cough	30.3	27.1	0.33
Influenza-like symptoms	39.8	35.9	0.25
Abdominal pain	23.6	25.2	0.67
Diarrhoea or obstipation	12.3	10.3	0.29
Allergy	7.8	11.3	0.14
Fatigue	45.8	45.4	0.88
Insomnia	23.2	21.6	0.59
Anxiety	14.4	13.2	0.59
Stress	30.3	31.1	0.87

## Appendix A

HepaMeta Team: Peter Jarčuška, Andrea Madarasová Gecková, Mária Mareková, Daniel Pella, Leonard Siegfried, Pavol Jarčuška, Lýdia Pastvová, Ján Fedačko, Jana Kollárová, Peter Kolarčik, Daniela Bobáková, Zuzana Veselská, Ingrid Babinská, Sylvia Dražilová, Jaroslav Rosenberger, Ivan Schréter, Pavol Kristian, Eduard Veselíny, Martin Janičko, Ladislav Virág, Anna Birková, Marta Kmeťová, Monika Halánová, Darina Petrášová, Katarína Cáriková, Viera Lovayová, Lucia Merkovská, Lucia Jedličková, Ivana Valková.
